# Rehabilitation needs and participation restriction in patients with cognitive disorder in the chronic phase of traumatic brain injury

**DOI:** 10.1097/MD.0000000000005968

**Published:** 2017-01-27

**Authors:** Hironobu Sashika, Kaoruko Takada, Naohisa Kikuchi

**Affiliations:** a Department of Rehabilitation Medicine, Graduate School of Medicine, Association of Medical Science; b Graduate School of Medicine, Yokohama City University, Kanazawa Ward; c Department of Rehabilitation Medicine, Yokohama City University Medical Center, Association of Medical Science, Yokohama City University, Minami Ward, Yokohama City, Japan.

**Keywords:** psychosocial problems, qualitative research, quality of life, rehabilitation, social participation, traumatic brain injury

## Abstract

The purpose of this study was to clarify psychosocial factors/problems, social participation, quality of life (QOL), and rehabilitation needs in chronic-phase traumatic brain injury (TBI) patients with cognitive disorder discharged from the level-1 trauma center (L1-TC), and to inspect the effects of rehabilitation intervention to these subjects.

A mixed-method research (cross-sectional and qualitative study) was conducted at an outpatient rehabilitation department.

Inclusion criteria of subjects were transfer to the L1-TC due to TBI; acute-stage rehabilitation treatment received in the L1-TC from November 2006 to October 2011; age of ≥18 and <70 years at the time of injury; a score of 0–3 on the Modified Rankin Scale at discharge and that of 4–5 due to physical or severe aggressive behavioral comorbid disorders. Study details were sent, via mail, to 84 suitable candidates, of whom 36 replied. Thirty-one subjects (median age: 33.4 years; male: 17; and average time since injury: 48.1 months), who had consented to study participation, were participated. Cognitive function, social participation, QOL, psychosocial factors/problems, rehabilitation needs, and chronic-phase rehabilitation outcomes were evaluated using the Wechsler Adult Intelligence Scale, Third Edition, the Wechsler Memory Scale-Revised, the Zung Self-Rating Depression Scale, the Sydney Psychosocial Reintegration Scale, Version 2, and the Short Form 36, Version 2, qualitative analysis of semistructured interviews, etc.

Participants were classified into achieved-social-participation (n = 11; employed: 8), difficult-social-participation (n = 12; unemployed: 8), and no-cognitive-dysfunction groups (n = 8; no social participation restriction). Relative to the achieved-social-participation group, the difficult-social-participation group showed greater injury and cognitive dysfunction and lower Sydney Psychosocial Reintegration Scale and Short Form 36 role/social component summary scores (64.9/49.1 vs 44.3/30.4, respectively, *P* < 0.05). Linear regression analysis showed that the social participation status was greatly affected by the later cognitive disorders and psychosocial factors/problems not by the severity of TBI. No changes were observed in these scores following chronic-phase rehabilitation intervention.

Chronic-phase TBI with cognitive disorder led to rehabilitation needs, and improvement of subjects’ psychosocial problems and QOL was difficult.

## 1 Introduction

According to the Centers for Disease Control and Prevention, 1,700,000 individuals sustained traumatic brain injury (TBI) annually in the USA between 2002 and 2006.^[1]^ Of those, 1,365,000 (80.7%) attended emergency departments, 275,000 (16.3%) were admitted and 52,000 (3.0%) died. There was no estimate for the number of people with nonfatal TBI who did not attend emergency departments.^[1]^ In 2010, the Centers for Disease Control and Prevention^[2]^ estimated that 2,500,000 individuals experienced TBI in the USA. Of these individuals, 2,213,826 (87%) were treated and released by emergency departments, 283,630 (11%) were hospitalized and discharged, and 52,844 (approximately 2%) died. The annual age-adjusted rate of total TBI-related emergency department visits, hospitalizations, and deaths has increased by 45%, from 566.7 to 823.7 per 100,000 persons from 2007 to 2010, in the USA.

Zaloshnja et al^[3]^ estimated that 3,170,000 people (95% CI: 3,020,000–3,320,000) in the USA were living with a long-term disability resulting from TBI at the beginning of 2005. In 2008, Watanabe et al^[4]^ estimated that in Tokyo (population: 12,800,000), 379 individuals sustained TBI involving cognitive dysfunction annually.

**Figure 1:**
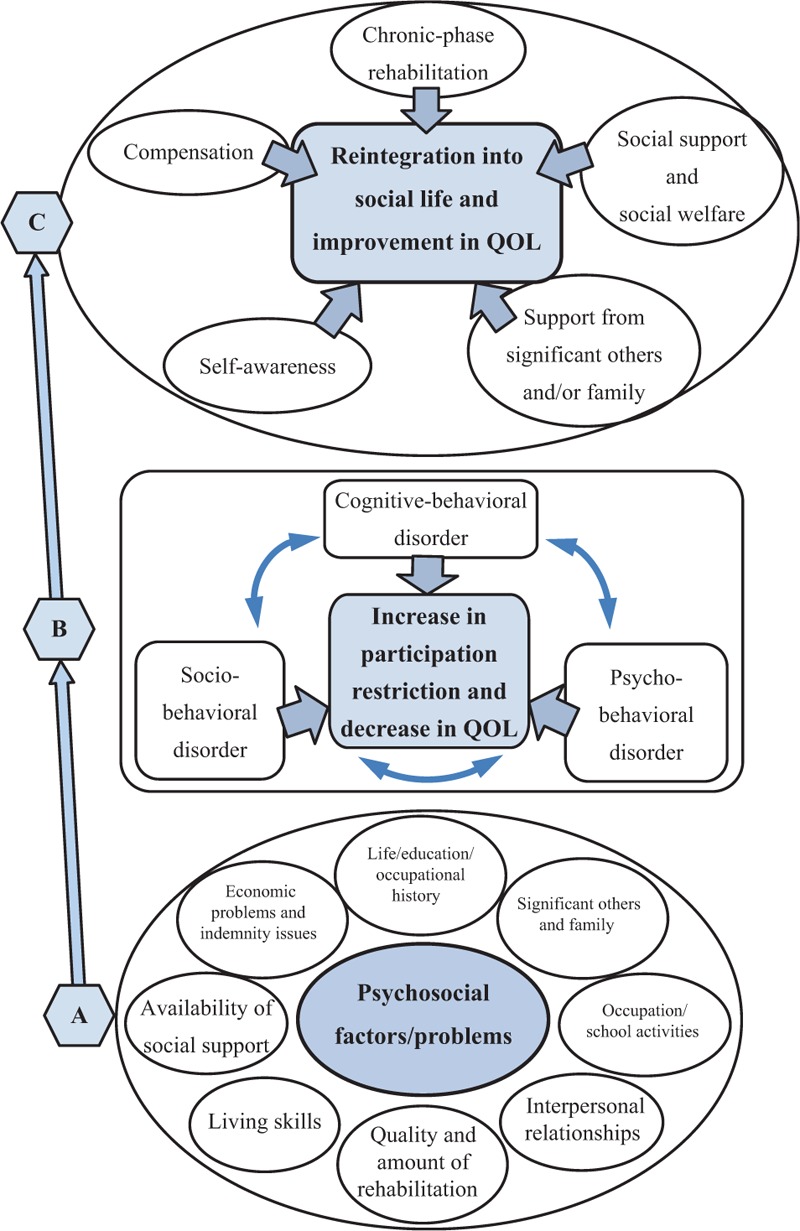
Psychosocial factors/problems in chronic phase TBI, participation restriction, and reintegration, (A) TBIs with cognitive dysfunction in the chronic phase; psychobehavioral and sociobehavioral disorders are affected by psychosocial factors/problems. (B) When rehabilitation and support for the cognitive-behavioral, psychobehavioral, and sociobehavioral disorders are insufficient, social participation is inhibited, and QOL decreases. (C) With self-awareness of cognitive disorder, social reintegration could be achieved with social support, adequate support from significant others, and chronic-phase rehabilitation. QOL = quality of life, TBI = traumatic brain injury.

Cognitive dysfunction occurs during TBI, but the functional disorders at the core of this dysfunction include executive dysfunction and memory, attention, cognitive-behavioral, and psychobehavioral disorders, which combine to create a plethora of sociobehavioral disorders. Furthermore, psychological and sociobehavioral disorders are affected by psychosocial factors such as preinjury developmental and lifestyle history, psychosocial problems related to postinjury social life, and the quality and amount of rehabilitation. If psychosocial problems are magnified, activity limitation, participation restriction, and quality of life (QOL) degradation increase (Fig. 1). Therefore, rehabilitation outcomes for cognitive dysfunction in TBI must include assessment that involves QOL and participation restriction and is not restricted to cognitive impairment and activity limitation.

However, there is no clear definition for psychosocial factors/problems. Generally, psychosocial factors/problems include issues involving interpersonal relationships, family members, school- and occupation-related problems, availability of social support, economic problems, and indemnity.

At the university hospital in Yokohama City, Japan, TBI patients are transported to the level-1 trauma center (L1-TC). Once patients have been stabilized, they are discharged home or transferred to a general, rehabilitation, or long-term care hospital. In the L1-TC, interventions involving acute-phase rehabilitation are initiated during the early “confused-agitated” or “confused, inappropriate, and nonagitated” stage^[5]^ following injury. In most cases, movement disorders or self-care issues are almost resolved at the time of transfer or discharge from the L1-TC, but cognitive screening tests performed by occupational therapists show that some type of cognitive impairment could be present.

Cognitive-behavioral, psychobehavioral, and sociobehavioral disorders do not commonly manifest during acute rehabilitation. During the chronic phase, psychosocial problems resulting from cognitive dysfunction manifest subsequent to discharge. However, individuals with TBI are often reluctant to visit rehabilitation departments and agree only after being urged to do so by important family members or significant others.

However, during the chronic stage, neurosurgery departments are the main providers of medical services. In addition, the rehabilitation provided in follow-up studies involving TBI patients who have been discharged from the hospital's emergency department or L1-TC is insufficient, and issues surrounding their social participation and QOL are not considered.

Rehabilitation outcomes typically involve either employment or domestic life. If the outcome is that the patient is unfit for occupational and domestic life, the use of social participation resources is inadequate, which could increase the risk of psychosocial problems such as social withdrawal at home. In addition, even when patients are employed, rehabilitation support should be offered at workplace. The lack or cessation of rehabilitation support could make continuing occupational activity difficult for patients.

The purpose of this study was to elucidate the psychosocial factors/problems of chronic-phase TBI patients who had been discharged from the L1-TC at the university hospital. A cross-sectional study involving mixed methods^[6]^ (quantitative and qualitative analysis) was designed. Semistructured interviews were conducted to determine the psychosocial factors/problems, social participation, QOL, and extent of subjects’ rehabilitation needs. Rehabilitation interventions were then implemented according to subjects’ rehabilitation needs, and intervention outcomes were evaluated using changes in social participation and QOL.

## 2 Methods

The study was conducted between June 2012 and March 2014.

### 2.1 Subject selection

**Figure 2:**
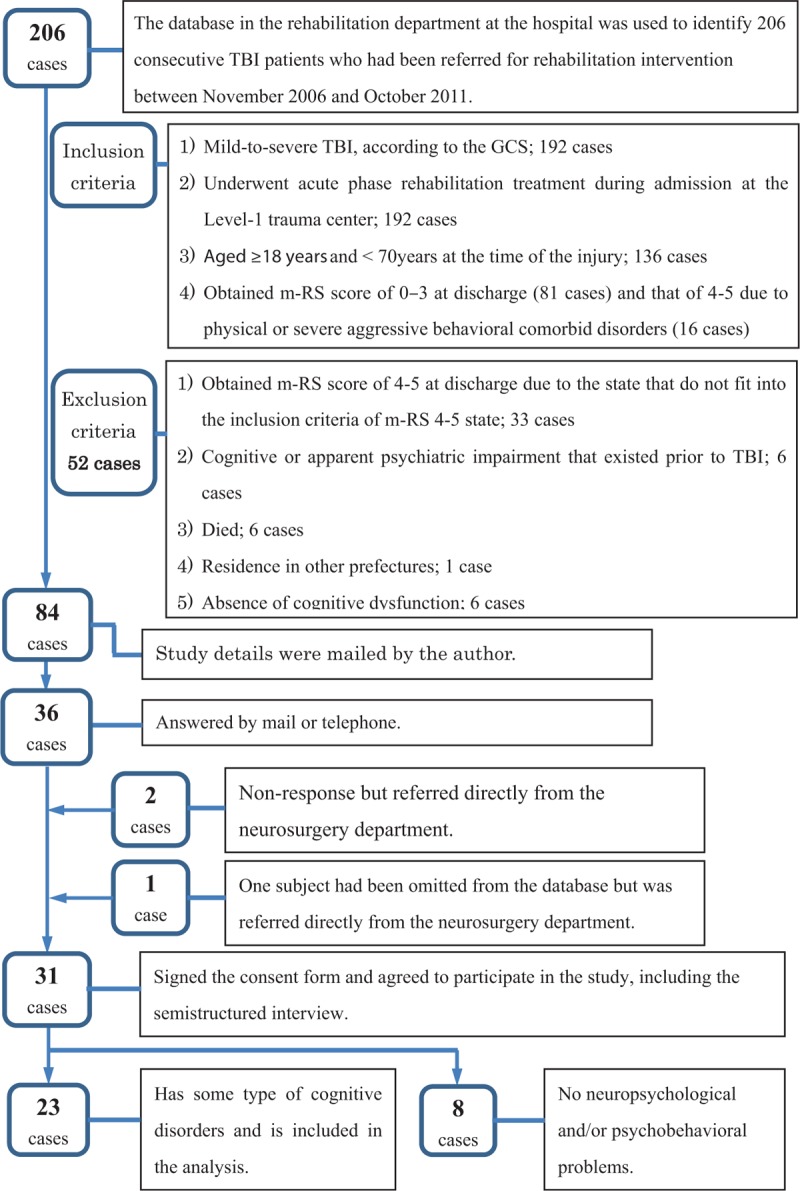
Subject selection flowchart, GCS = Glasgow coma scale, m-RS = modified Rankin Scale, TBI = traumatic brain injury.

Figure 2 depicts the subject selection flowchart.

In the 1st step, the database at the university hospital rehabilitation department was used to identify 206 consecutive TBI patients who had been referred for rehabilitation intervention between November 2006 and October 2011.

Patients’ medical information pertaining to the L1-TC was collected from the hospitalization medical records department at the L1-TC.

The 2nd step involved application of the following inclusion criteria: mild to severe TBI, according to the Glasgow coma scale (GCS), and transfer to the L1-TC (192 cases); acute-phase rehabilitation treatment received in the L1-TC (192 cases); age of ≥18 years and <70 years at the time of TBI (136 cases); and a score of 0–3 on the Modified Rankin Scale (m-RS) at discharge (81 cases) and that of 4–5 at discharge due to physical or severe aggressive behavioral comorbid disorders (16 cases).

The exclusion criteria were: a score of 4–5 on the m-RS at discharge due to the state that do not fit into the above-mentioned inclusion criteria of m-RS 4–5 state (33 cases); cognitive or apparent psychiatric impairment that existed prior to TBI (6 cases); died (6 cases); residence in other prefectures (1 case); and absence of cognitive dysfunction (6 cases).

Consequently, 52 patients were excluded according to the exclusion criteria from 136 patients and 84 suitable candidates were selected.

In the 3rd step, study details were sent, via mail, to 84 suitable candidates, of whom 36 replied. Three additional candidates were referred directly from the neurosurgery department (1 subject had been omitted from the database). Of 39 potential subjects, 31 consented in a document to study participation.

### 2.2 Qualitative data collection in the 1st interview; assessment of psychosocial factors/problems

The qualitative data were collected mainly via semistructured interviews. Of available qualitative approaches,^[7]^ the modified grounded theory approach developed by Kinoshita is the most popular grounded theory approach in Japan.^[8]^ Modified grounded theory approach amendments intended to clarify the coding method were made following application of the basic properties of original grounded theory (intentionality to theory generation, principle of grounded-on data, empiricism and positivism, and practical application as a viewpoint of verification).^[9]^

Two rehabilitation doctors, who were certified specialists of the Japanese Association of Rehabilitation Medicine, conducted the initial intake and semistructured interviews with 31 subjects at home or in an interview room at the rehabilitation department. The interviewer requested family members or significant others to attend the interviews whenever possible.

During the semistructured interviews, subjects could answer the following questions freely: “What are the conditions of your current lifestyle, occupation, friends, and family;” “What are the residual TBI effects that you currently experience;” “Which 3 incidents in your life have been influenced most by TBI, in consecutive order;” and “What are your hopes for rehabilitation?” Interviews were transcribed verbatim using shorthand. The main interviewer documented the content of conversations on the day of the interview, and another interviewer confirmed whether this content was accurate.

Descriptive content regarding subjects’ psychosocial factors/problems, social participation restriction, QOL, rehabilitation needs, and occupational or educational status was extracted using the interview transcripts. Following application of the modified grounded theory approach coding method,^[9]^ concept formation and category generation were performed.

### 2.3 Quantitative data collection at the 1st interview; assessment of cognitive dysfunction, psychological disorders, social participation restriction, and QOL

Assessment of cognitive dysfunction: The Wechsler Adult Intelligence Scale, Third Edition (WAIS-III), Wechsler Memory Scale-Revised (WMS-R), Cognitive-Behavior Scale for TBI,^[10]^ Behavioral Assessment of the Dysexecutive Syndrome (BADS), and Rivermead Behavioral Memory Test (RBMT)^[11]^ were used to assess cognitive dysfunction, regarding which information was also obtained from recent data held at rehabilitation hospitals implementing follow-up.

The Cognitive-Behavior Scale for TBI^[10]^ is a self-assessment questionnaire consisting of 31 items used in cognitive dysfunction screening. Seven factors, including amnesia (9 items) and executive function (4 items), are extracted through factor analysis. The standard deviation for control subjects is used as the measurement unit, with scores for each factor expressed as z values and healthy individuals’ mean scores for each factor designated as the zero point. The higher a subject's z score, the more severe the impairment. In this study, the amnesia factor (9 items) was included in the analysis.

The total score of RBMT are 24 point. The original English version of RBMT^[11]^ used the cut-off point according to the level of memory disturbance; 22–24 in normal, 17–21 in poor memory, 10–16 in moderately impaired, and 0–9 in severely impaired.

Assessment of psychological disorders: The Zung Self-Rating Depression Scale^[12]^ (SDS) was used to assess depressive state. The SDS is a 20-item self-assessment questionnaire that measures depression. Item scores range from 1 to 4 (total score range: 20–80). In the Japanese edition of the SDS, scores of 40–49 and ≥50 indicate depressive state and depression, respectively.

Assessment of social participation restriction: The Sydney Psychosocial Reintegration Scale, Version 2 (SPRS-2)^[13,14]^ was used to assess social participation restriction. The SPRS-2 consists of 12 items concerning work and leisure activities, interpersonal relationships, and independent living skills. Higher scores indicate favorable social participation. If total scores (0–48) are converted to logit values (0–100 logit) using Rasch analysis, no ceiling or floor effects are observed. The reliability and validity of the scale have been demonstrated statistically. Form A (physician-led assessment questionnaire) measures change since injury, and form B (self-assessment questionnaire) measures current status. Because there is no Japanese version of the scale, the SPRS-2 (forms A and B) was translated into Japanese for use in the study.

Assessment of QOL: The Japanese edition of the 2nd version of the 36-Item Short-Form Health Survey, or Short-Form 36 (SF-36v2),^[15,16]^ was used to assess QOL. The SF-36 is a 36-item health-related QOL measure that was developed in the USA; the reliability, validity, and national standard value (norm-based scoring) of the scale have been demonstrated. The SF-36v2 contains 8 domains, for which scores range from 0 to 100; higher scores indicate better QOL. Physical, mental, and role/social component summary scores are calculated based on the scores for the 8 domains. Norm-based scoring (mean score: 50, standard deviation: 10) is published according to sex and age. Furthermore, the SF-36v2 was used to assess the effect of subjects’ cognitive dysfunction on significant others.

### 2.4 Prospective rehabilitation intervention based on clarified rehabilitation needs

Content related to subjects’ social participation and rehabilitation needs was extracted from the semistructured interview transcripts and classified into concepts or categories concerning social participation and rehabilitation needs.

Consent for prospective chronic-phase rehabilitation intervention was obtained from the subjects, and the services were implemented based on subjects’ specific rehabilitation needs.

### 2.5 Additional assessments 12 to 18 months subsequent to interviews

Approximately 12 to 18 months subsequent to the initial interviews, an additional self-assessment questionnaire measuring participation restriction and rehabilitation needs and including the SPRS-2 (Form B) and SF-36v2 was sent to subjects via mail. The self-assessment questionnaire items were intended as follow-up for those used in the interviews. Scores for the SPRS-2, SF-36v2, and variables, such as social participation, included in the additional assessment were compared to those of the initial interviews.

Rehabilitation intervention outcomes were assessed according to differences between the scores for variables in the initial interview and additional assessment. Relative to that recorded at initial interview, QOL at additional assessment was categorized as improvement, no change, decline, or breakdown of the life.

### 2.6 Statistical analysis and ethical considerations

The sample size was calculated to examine changes in SF-36v2 scores. With regard to norm-based scoring for the Japanese population, the mean SF-36v2 score was 50 (standard deviation: 10). Therefore, the required sample size for the independent *t* test was 17 (α: 0.05, power: 0.8, δ: 10, and σ: 10).

IBM SPSS Statistics version 21 was used for statistical analysis. A *t* test, analysis of variance, and a chi-square (χ^2^) test were performed in the statistical analysis. A stepwise linear regression analysis was conducted. Dependent variables were social participation status, Sf-36v2, and SPRS-2 score at the initial interview. Independent variables were 23 items/domains such as education years, TBI severity (GCS score), cognitive function (WAIS-III), psychological disorders (SDS), psychosocial factors, QOL (SF-36v2), etc. In order to confirm the validity of the participant selection, Cronbach-alfa was calculated. A *P* value of <0.05 was considered statistically significant.

The clinical ethical review board at the university with which the authors were affiliated approved the study in June 2012. This study was conducted in accordance with the ethical standards established in the Declaration of Helsinki (1964) and later amendments.

## 3 Results

### 3.1 Subject characteristics

Thirty-one subjects participated in the interviews. Cronbach-alfa test for 8 subdomains of SF-36v2 and 12 items of SPRS-2 were 0.832 and 0.963, respectively. Subject characteristics are presented in Table 1.

**Table 1:**
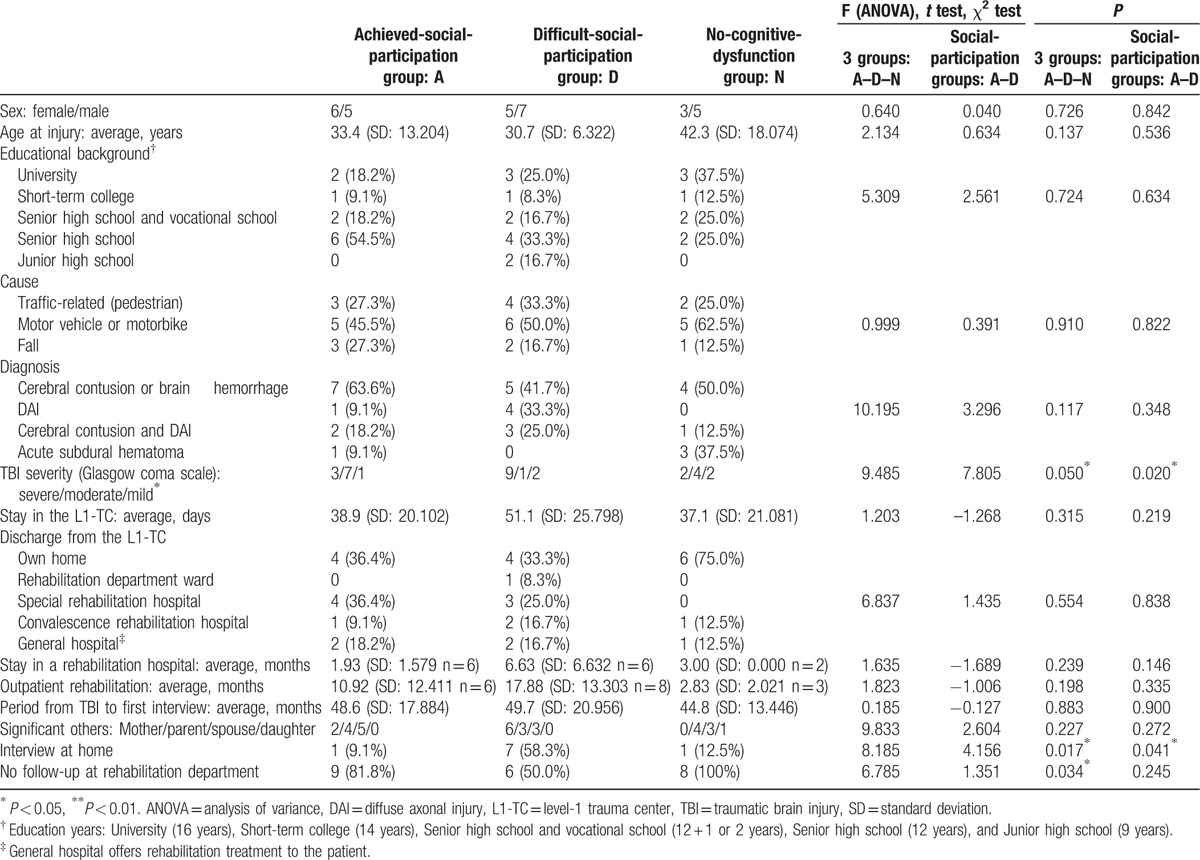
Subjects’ basic information at the initial interview.

The content of the initial intake and semistructured interviews was clear and logical for 8 subjects, who did not show memory disturbance or attention disorder in everyday life or display participation restriction in their work, school, or community lives. These 8 subjects did not display cognitive dysfunction and were assigned to the group without cognitive dysfunction (no-cognitive-dysfunction group). They were subsequently excluded from the analysis and the chronic-phase rehabilitation intervention.

In the 11 subjects who showed cognitive dysfunction, social participation levels ranged from good (full-time employment, student, or independent housewife) to inadequate (part-time employment or partially dependent housewife). These subjects were assigned to the group that achieved social participation despite cognitive disorder (achieved-social-participation group).

For 12 subjects, social participation was difficult (institutional support for daily life activity or employment) or impossible (entirely dependent housewife, unemployed). These subjects were assigned to the group that experienced difficult social participation (difficult-social-participation group).

Participants’ median age at injury was 33.4 years (range: 18.3–62.9 years, *P* = 0.137), with injury most common between youth and middle age. The educational background did not show significant difference between 3 groups statistically (*P* = 0.724). Most subjects from all 3 groups had suffered trauma in a traffic accident; all 3 groups included individuals classified as having severe TBI (GCS: 3–8) upon transfer to the L1-TC.

Diffuse axonal injury was observed in 58% of the difficult-social-participation group, which also included 9 subjects with severe TBI; this number exceeded that of similar subjects in the achieved-social-participation group (*P* = 0.020). The duration of the L1-TC hospitalization was long (median: 40 days) for all groups; this was particularly true for the difficult-social-participation group. Forty-five percent of achieved-social-participation group and 50% of the difficult-social-participation group subjects were subsequently transferred to a convalescence rehabilitation hospital or special rehabilitation hospital for TBI. Outpatient rehabilitation period of the difficult-social-participation group (n = 8) was long. Despite experiencing cognitive dysfunction, 9 (81.8%) and 6 (50.0%) subjects from the achieved- and difficult-social-participation groups, respectively, did not receive follow-up treatment from the rehabilitation department (*X*^2^ = 1.351, *P* = 0.245), which was also true for all no-cognitive-dysfunction group subjects (F = 6.758, *P* = 0.034).

The median period from TBI to 1st interview was 50.7 months (range: 14–82 months, *P* = 0.883).

The results obtained for the achieved- and difficult-social-participation groups are described below.

### 3.2 Psychosocial factors/problems extracted from initial intake and semistructured interviews

Numerous concepts and categories were extracted from verbatim interview transcripts; of these, psychosocial factors/problems that could have influenced subjects’ social participation restriction, QOL, and rehabilitation needs were analyzed qualitatively. Pre-TBI psychosocial factors are shown in Table 2.

**Table 2:**
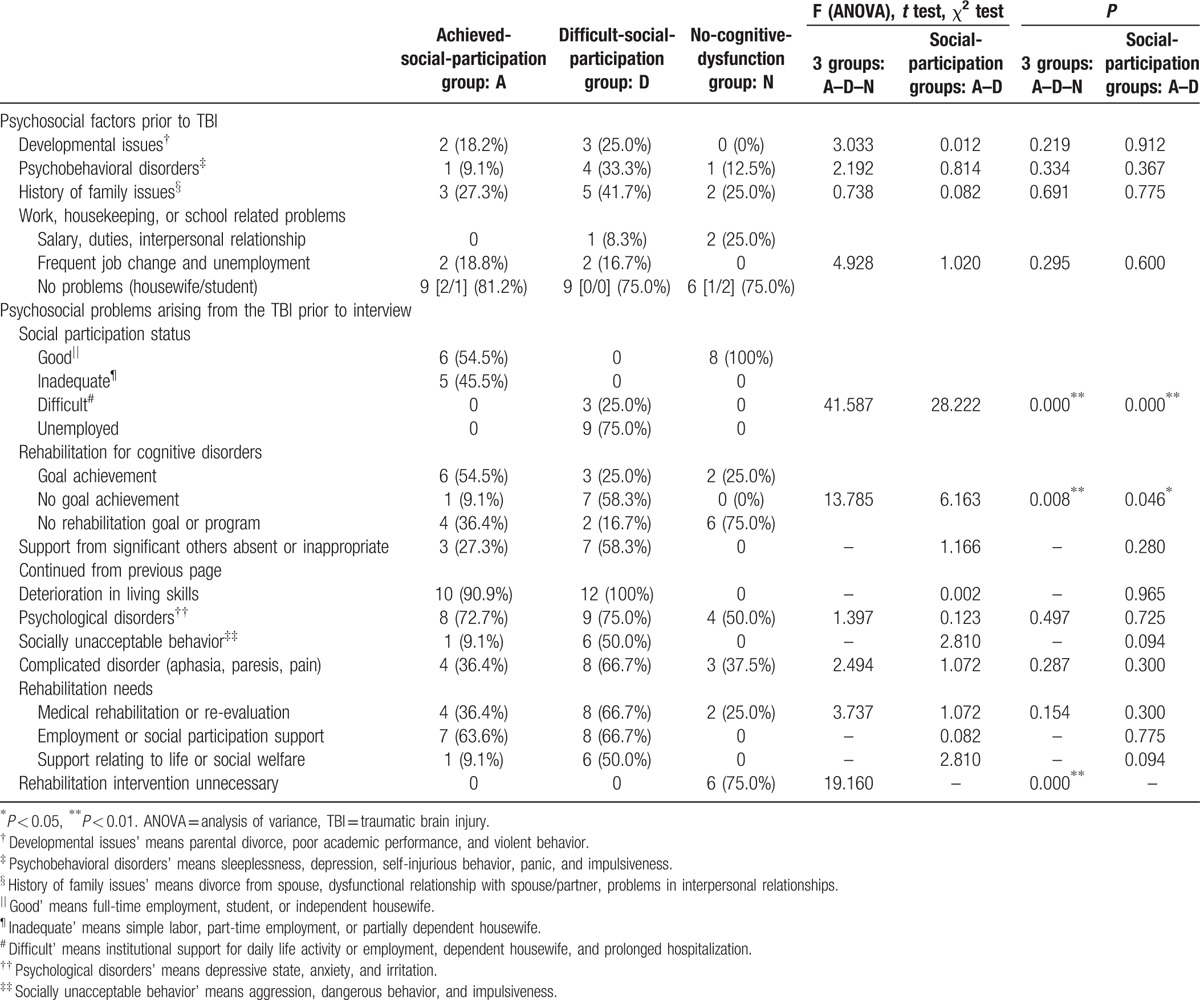
Psychosocial factors/problems extracted from initial intake and semistructured interviews: psychosocial factors/problems, participation restriction, quality of life, rehabilitation needs, and occupation.

Approximately 20% of subjects in both social-participation groups showed a history of developmental issues such as parental divorce and poor academic performance. In addition, 33.3% of the difficult-social-participation group subjects exhibited psychobehavioral disorders such as sleeplessness, depression, self-injurious behavior (with wrist scars indicating self-cutting), and panic. Approximately 27% and 42% of achieved- and difficult-social-participation group subjects, respectively, reported a history of family issues such as divorce. Moreover, 19% and 25% of achieved- and difficult-social-participation group subjects, respectively, reported work-related problems, with no significant between-group difference (χ^2^ = 1.020, *P* = 0.60).

Psychosocial problems that arose during the period between TBI and initial interviews are shown in Table 2. Six (54.5%) and 3 (25.0%) achieved- and difficult-social-participation group subjects, respectively, achieved their rehabilitation goals for cognitive dysfunction. In both groups, more than 70% of subjects reported associated psychological disorders such as anxiety. In the difficult-social-participation group, 50% of subjects showed socially unacceptable behavior such as aggression, violation of a social rule, dangerous behavior, or impulsiveness.

Seven (63.6%) and 8 (66.7%) achieved- and difficult-social-participation group subjects, respectively, exhibited rehabilitation needs involving social participation such as occupational support. In both groups, all subjects required intervention via rehabilitation.

### 3.3 Cognitive function, psychological disorders, and QOL: initial evaluation at 1st interview

Relative to the achieved-social participation group, the difficult-social-participation group obtained significantly lower WAIS-III (performance intelligence quotient), WMS-R (Auditory Index and Delayed recall Index), and BADS scores and higher Cognitive-Behavior Scale for TBI amnesia scores (Table 3).

**Table 3:**
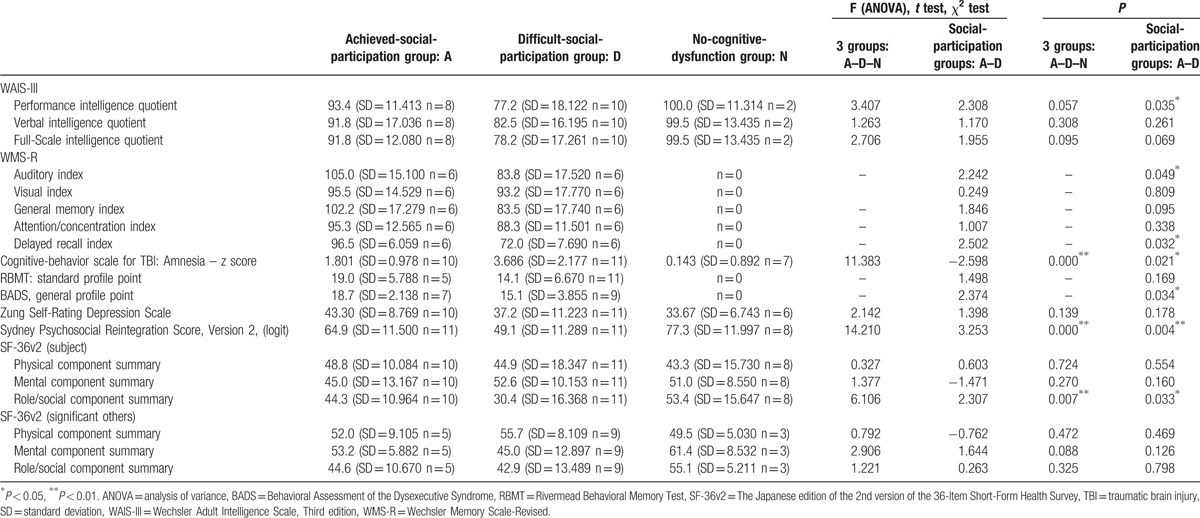
Examination of neuropsychological functions, psychological disorders, quality of life, and social participation: initial evaluation at initial interview.

SDS scores for the achieved- and difficult-social-participation groups were somewhat high but did not differ significantly (*t* = 1.398, *P* = 0.178).

SPRS-2 scores (form A) were significantly lower in the difficult-social-participation group relative to those of achieved-social-participation group (*t* = 3.253, *P* = 0.004).

Furthermore, regarding QOL, the difficult-social-participation group obtained significantly lower SF-36v2 role/social component summary scores relative to those of the achieved-social-participation group (*t* = 2.307, *P* = 0.033). Further, SF-36v2 scores related to significant others did not differ significantly between the 2 groups.

### 3.4 The association with TBI and the psychosocial factors/problems

The results of the stepwise linear regression analysis are shown in Table 4. Dependent variable of “Social participation status at 1st interview” was associated with the performance intelligent quotient of WAIS-III and the role/social component summary of SF-36v2. On the other hand, 3 component summaries of SF-36v2 and SPRS-2 mainly associated with sex, education years, social participation, psychosocial factors/problems, psychological status, etc.

**Table 4:**
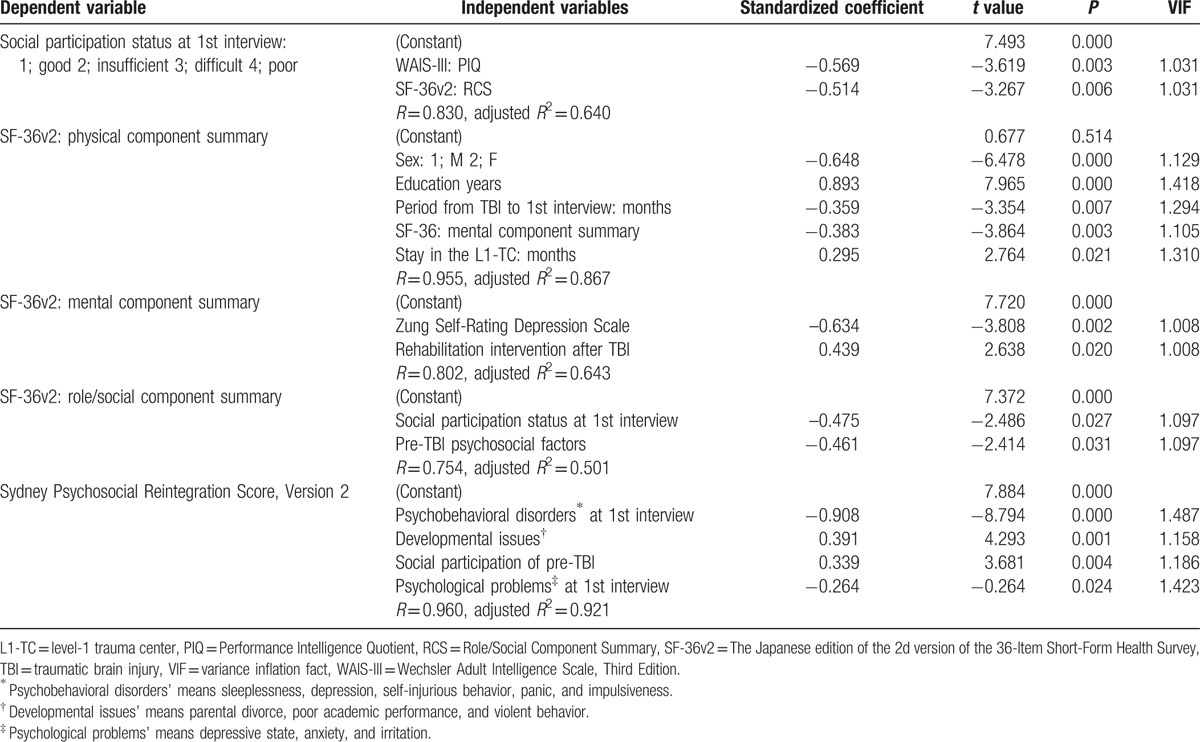
Results of the stepwise linear regression analysis.

### 3.5 Rehabilitation interventions and outcomes

Details of prospective chronic-phase rehabilitation interventions performed during the period between interviews and additional assessments are shown in Table 5. Nine and 11 achieved- and difficult-social-participation group subjects, respectively, engaged in prospective rehabilitation interventions based on their rehabilitation needs.

**Table 5:**
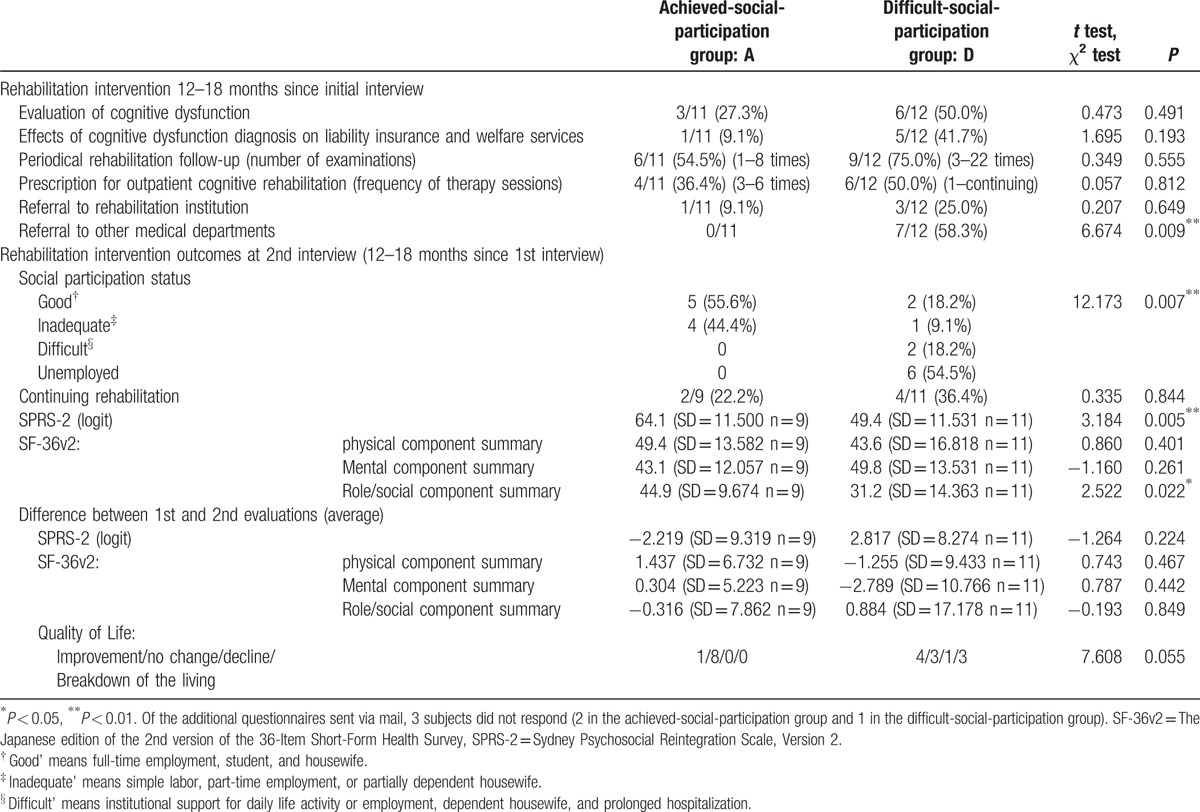
Rehabilitation intervention and outcomes in the period between the interview and additional assessment.

In the achieved-social-participation group, interventions included periodic rehabilitation follow-up (6 subjects), outpatient rehabilitation for cognitive dysfunction (4 subjects), and others. In the difficult-social-participation group, interventions included cognitive dysfunction evaluation (6 subjects), outpatient rehabilitation for cognitive dysfunction (6 subjects), referral to the rehabilitation institution for social participation promotion (3 subjects), and others. However, because the Japanese healthcare insurance system has not adopted holistic day treatment rehabilitation, the program was not offered.

Of the subjects who were referred to the rehabilitation institution, 2 underwent consultation. Of these, 1 withdrew prior to initiation of an employment support program. Another subject, who had experienced repeated dismissal subsequent to TBI injury, secured employment independently via the Internet and withdrew from the employment support program prior to completion. These subjects no longer underwent follow-up at the rehabilitation institution.

Regarding the outcomes of the 12 to 18-month prospective rehabilitation intervention, in the difficult-social-participation group, social participation improved slightly and the number of unemployed subjects decreased from 9 (75%; Table 2) at initial interview to 6 (54.5%) at additional assessment. At both initial interview and additional assessment, the difficult-social-participation group displayed significantly lower SPRS-2 score and SF-36v2 role/social component summary scores relative to those of the achieved-social-participation group. However, neither group experienced significant changes in these scores subsequent to the rehabilitation intervention.

At additional assessment, 11.1% and 36.4% of achieved- and difficult-social-participation group subjects, respectively, showed improved QOL, but significant difference between both groups was not shown (*X*^2^ = 7.608, *P* = 0.055). Three subjects from the difficult-social-participation group were considered to have experienced a breakdown of the living.

### 3.6 Presntation of 2 participants who had category of the “risk of maintaining social participation (employment)” and “ambiguous rehabilitation needs”

Case 1: Male (age at injury; 18 years old, motorcycle accident).

Parents were divorced during a high school student. He was a part-time worker after graduation from senior high school. He was injured for brain contusion (a bilateral frontal-temporal lobe) and diffuse axonal injury. GCS at the time of the L1-TC import was E1V2M2. Length of stay in the L1-TC was 43 days. He was transferred to TBI rehabilitation hospital, and discharged to mother's house after 153 days from injury.

Rehabilitation goal was “social welfare work,” but he employed as a handicapped worker to a company for handicapped (labor days; 14 days in 1 month). Follow-up by rehabilitation department was not done.

His self-awareness for moderate cognitive dysfunction (memory disturbance, etc.) was insufficient. His psychobehavioral disorders were making excuses and lie for his failure, easiness of anger (beating things “bang, bang”), poor managing money or life, etc.

Rehabilitation needs of mother and the social worker was to have rehabilitation again, and that of him was vague hope to living alone. He was pressed mother and a social worker for visiting the rehabilitation medicine 54 months after the injury. [WAIS-III; PIQ 82 VIQ 86 FIQ 83, BADS 19].

He answered in the initial interview; “I want my mother not to say ‘You could do it before’.” “Colleagues say to me ‘Work is slow, and to do that work than the work earlier’.” “I can intend to quit work. I am working but do not think it is ‘good.”

Case 2: Male (age at injury; 22 years old, motorcycle accident).

Parents were divorced at a primary schoolboy. He was a national public servant (a round of inspection and supervisory duties) after graduation from senior high school. He was injured for brain contusion (a left frontal-temporal lobe) and multiple bone fractures such as the fracture of orbit. GCS at the time of the L1-TC import was E1V2M4. Length of stay in the L1-TC was 60 days. He was transferred to a general hospital of the hometown. He discharged the hospital to mother's house in 72 days from the injury, and then continued outpatient cognitive rehabilitation for 4 months. He returned to an office work in 13 months from the injury, but there were no rehabilitation follows afterward. He completely returned to his former duties in 43 months.

After injury in 48 months, he consulted the rehabilitation medicine for the purpose of aftereffect diagnosis. The rehabilitation needs to the cognitive disorder was vague.

He was aware of his memory disturbance and difficulty of handling multiple tasks. Memory disturbance was remarkable, but the recent memory was kept. The psychobehavioral disorder such as easiness of anger or impulsiveness was not found. [WAIS-III; PIQ 92 VIQ 85 FIQ 87, BADS 16].

He could input a promise and a schedule into i-phone promptly. He was going to hide cognitive disorders from a boss and fellow workers. Therefore, he was not able to talk about his cognitive disorders in the workplace.

He answered in the initial interview; “In patrolling work, observations to a wide range cannot be reached every corner.” “I forget the place where I put a memo for the patrol report.” “I will panic when I receive various instructions continuously.” “My boss strictly warned me what I forgot and failed.”

## 4 Discussion

The pathways of TBI patients discharged from an L1-TC in The Netherlands were studied retrospectively by de Koning et al.^[17]^ The results showed that 62% and 22% of subjects with moderate (GCS: 9–12, n = 89) and severe TBI (GCS: 3–8, n = 254), respectively, were initially discharged home. Most subjects (94%), including those who were discharged via rehabilitation hospitals, general hospitals, and nursing homes, had returned home 1 year subsequent to injury (98% and 92% with moderate and severe TBI, respectively). One year subsequent to injury, 1 in 4 patients exhibited cognitive disorders, behavioral disorders, and physical disabilities, with only 32% able to resume their preinjury occupations. Almost half of TBI patients needed outpatient care for cognitive-behavioral disorders. Of those initially discharged home without follow-up, 10% subsequently required outpatient rehabilitation. The researchers concluded that long-term aftercare for chronic-phase TBI was required to improve social participation.^[17]^

The qualitative findings of the present study showed that, in the chronic stage of TBI, cognitive-behavioral disorder fluctuated according to psychosocial issues. TBI patients with cognitive dysfunction sometimes discontinued the rehabilitation department's serial observation inappropriately after returning home and engaging in social participation involving activities such as working. Subjects or their significant others subsequently become aware of cognitive-behavioral disorders and the need for chronic-phase rehabilitation. Results of the stepwise linear regression analysis showed that the social participation status in the chronic phase TBI was greatly affected by the later cognitive disorders (WAIS-III; performance intelligence quotient) and psychosocial factors/problems not by the TBI severity (GCS).

Cognitive-behavioral and psychobehavioral disorders observed in chronic-phase TBI were affected by psychosocial factors/problems and tended to manifest gradually. When rehabilitation and support for these disorders were insufficient, social participation was inhibited and QOL decreased. In contrast, with self-awareness of cognitive disorder, social reintegration could be achieved with social support, adequate support from significant others, and chronic-phase rehabilitation.

Because of TBI patients’ impaired memory and awareness, the reliability of the content of their responses to self-assessment questionnaires should be considered. Sherer et al^[18]^ systematically reviewed the prognostic value of self-reported traits/problems/strengths and environmental barriers/facilitators related to TBI patients’ participation outcomes. Of the self-reported variables, the number of postconcussive symptoms, fatigue, and physical competence were predictive of employment and need for supervision, whereas self-efficacy was unlikely to predict employment. Subjective well-being, pain, and social interaction, but not coping style, were possibly predictive of employment. The researchers concluded that self-report variables were likely to contribute to participation outcome prediction.

The present study involved self-assessment via the Cognitive-Behavior Scale for TBI, SPRS-2 (Form B), and SF-36v2, but the objectives of the study did not include identification of outcome predictors or observation of disparities between subjects’ self-assessment and evaluation performed by their significant others. To minimize the influence of subjects’ under- and overestimation, information provided by significant others was reviewed, semistructured interview transcripts were considered, and concepts and categories related to psychosocial factors/problems, social participation, QOL, and rehabilitation needs were extracted. Accordingly, the concepts and categories identified by the self-assessment questionnaires and semistructured interviews presumably exerted little influence on outcomes, despite errors in subjects’ self-assessment.

Generally, psychosocial factors include issues involving family members, interpersonal relationships, school and occupational issues, availability of social support, economic problems, and compensation. However, the definition and range of psychosocial factors remain unclear. Bond^[19]^ assessed psychosocial TBI outcomes using neurophysical, mental, and social scales.

Social participation and community integration, which are affected by psychosocial issues, are rehabilitation goals for TBI patients. Salter et al^[20]^ reviewed current approaches to the community integration assessment. The Community Integration Questionnaire, SPRS-2, Reintegration to Normal Living Index, and Community Integration Measure were used to assess community integration. In these measures, the 3 core elements of community integration included relationships with others, independence, and meaningful activities. Only the SPRS-2 assessed both postinjury changes (form A) and current status (form B), which were well correlated.^[14]^

Cattelani et al^[21]^ systematically reviewed neurobehavioral rehabilitation programs for adults with TBI and made evidence-based recommendations for program adoption. The researchers concluded that the greatest overall improvement in psychosocial functioning was achieved via comprehensive-holistic rehabilitation programs, which could be considered a treatment standard for TBI patients with behavioral and psychosocial disorders. Cicerone et al^[22]^ systematically reviewed cognitive rehabilitation in individuals with TBI and stroke. They made evidence-based recommendations for practice standards and provided evidence to support interventions to improve attention, memory, social communication skills, and executive function and the implementation of comprehensive-holistic neuropsychological rehabilitation following TBI.

Ben-Yishay et al^[23,24]^ developed and implemented the comprehensive-holistic day-treatment rehabilitation program for chronic TBI. In a review, Malec and Basford^[25]^ found that the program involved neuropsychological orientation, integrated treatment, group intervention, dedicated resources, a neuropsychologist as a team member, participation of significant others, dedicated vocational or independent living, and evaluation of multifaceted results. However, the program is seldom offered in Japan, because it has not been adopted by the healthcare insurance system. Therefore, in its modified form, the program should be offered as standard cognitive rehabilitation for individual issues in cognitive disorders. Therefore, if subjects’ exhibit little or no awareness of deviation in their social behavior (cognitive-behavioral disorder), they experience difficulty in solving complicated psychosocial problems and maintaining social participation involving activities such as work, increasing their risk of breakdown of the living.

The effect of chronic-phase rehabilitation intervention was insufficient in this study; this may have occurred because a comprehensive-holistic day treatment rehabilitation program was not offered.

### 4.1 Study limitations and challenges

Cultural and social differences between Japan and Australia should have been considered when translating the original English SPRS-2 into Japanese. However, because this would have required additional research, and the sample size was small, these differences were not explored.

In addition, the actual number of subjects did not match the sample size. Because this was a mixed-methods study involving qualitative data collection, it was difficult to perform a clinical study involving numerous subjects. Nevertheless, the number of subjects should be increased to reduce type I and II errors in future.

It was difficult to obtain accurate information in a single semistructured interview with TBI patients with cognitive dysfunction, because they engaged in circumlocution and deviated from the point. In particular, statements of participants relating to rehabilitation needs were often some ambiguity. Therefore, for confirming their rehabilitation needs, it was necessary to check the verbatim record or to conduct re-interview in some cases. This necessitated the repetition of interviews, which increased the possibility of bias such as that involving interviewers’ opinions.

In qualitative studies involving conversational data, it is important to use study methods that minimize bias in the analyst. Sufficient discussion regarding mismatched concepts/categories and computer software was necessary.

## 5 Conclusions

Even if initial TBI severity was severe, some subjects experienced no residual cognitive dysfunction or social participation restriction. Relative to the achieved- and difficult-social-participation group, the difficult-social-participation group included a significantly higher number of subjects with severe TBI (GCS: 3–8) and cognitive dysfunction in the chronic phase. Furthermore, there was a decrease in social participation and QOL measured via the SPRS-2 and SF-36v2. The social participation status in the chronic phase TBI was greatly affected by the later cognitive disorders (WAIS-III; performance intelligence quotient) and psychosocial factors/problems not by the TBI severity (GCS). There were no significant differences in QOL in subjects’ significant others in either group; therefore, they did not appear to be strongly affected by subjects’ cognitive dysfunction. Despite experiencing cognitive dysfunction, the achieved-social-participation group tended to discontinue their follow-up participation at the rehabilitation department. In addition, psychological disorders, such as anxiety, were observed in 70% of subjects, regardless of the extent of their social participation. Further, irrespective of the severity of social participation restriction, most subjects exhibited rehabilitation needs, and rehabilitation intervention was required in the chronic-phase of TBI. During this phase, the provision of supportive outpatient rehabilitation programs and continued follow-up at the rehabilitation department were important in social participation maintenance and enhancement, despite the fact that cognitive function did not improve in practice.

## Acknowledgments

The authors thank to Hidetaka Wakabayashi, MD (Yokohama City University Medical Center, Association of Medical Science, Yokohama City University) for statistical advice. The authors also thank Grant-in-Aid for Scientific Research (C) from the Japanese Ministry of Education, Culture, Sports, Science, and Technology: “A qualitative study of rehabilitation needs and participation restriction of chronic-phase subjects with cognitive disorder due to traumatic brain injury” (2012–2014), Project Number: 24500599 for the support.
